# Imaging of the Finger Vein and Blood Flow for Anti-Spoofing Authentication Using a Laser and a MEMS Scanner

**DOI:** 10.3390/s17040925

**Published:** 2017-04-22

**Authors:** Jaekwon Lee, Seunghwan Moon, Juhun Lim, Min-Joo Gwak, Jae Gwan Kim, Euiheon Chung, Jong-Hyun Lee

**Affiliations:** 1 School of Mechanical Engineering, Gwangju Institute of Science and Technology (GIST), Gwangju 61005, Korea; jkwon19@gist.ac.kr (J.L.); msh@gist.ac.kr (S.M.); 2 Department of Biomedical Science & Engineering, Gwangju Institute of Science and Technology (GIST), Gwangju 61005, Korea; johnlim@gist.ac.kr (J.L.); jaekim@gist.ac.kr (J.G.K.); ogong50@gist.ac.kr (E.C.); 3 Fiber Optic Electronic R&D Center, Korea Optron Corporation (KOC), Gwangju 61007, Korea; gmj87@naver.com

**Keywords:** infrared imaging, MEMS scanner, speckle imaging, transmission

## Abstract

A new authentication method employing a laser and a scanner is proposed to improve image contrast of the finger vein and to extract blood flow pattern for liveness detection. A micromirror reflects a laser beam and performs a uniform raster scan. Transmissive vein images were obtained, and compared with those of an LED. Blood flow patterns were also obtained based on speckle images in perfusion and occlusion. Curvature ratios of the finger vein and blood flow intensities were found to be nearly constant, regardless of the vein size, which validated the high repeatability of this scheme for identity authentication with anti-spoofing.

## 1. Introduction

Among the available security authentication technologies, biometrics, which involves identification via a body part, has been used in several fields, such as personal identification in banks and public offices [1,2,3]. Biometric approaches, including fingerprint, iris, retina, or voice recognition [2,3,4], do not require memorization of information, such as passwords or social security numbers. Fingerprint recognition remains popular due to its simplicity [4]. While recognition problems caused by sweaty fingers and/or degradation of fingerprints have been solved to a considerable extent, fingerprints are still exposed to the danger of counterfeiting. In particular, the optical coherence tomography (OCT) images of internal fingerprints and sweat pores inside fingerprints were demonstrated to prevent fingerprint spoofing at the price of their complexity and expensiveness [5].

To solve the aforementioned problems, a unique finger vein pattern can be used to authenticate a person more accurately, regardless of their change of fingerprint status [6,7,8,9,10,11]. Recently, finger vein imaging technology has been under development using the shadow effect of near-infrared light-emitting diodes (NIR LEDs) [6,7,8,9]. This method, however, might degrade the image contrast because LED light is not collimated and resultantly spreads out of the finger, which leads to higher background noise. Direct contact of the finger with the LEDs can enhance resolution, but may cause cross-contamination [10]. Meanwhile, the detection of blood flow in the finger vein is very important for liveness detection. Note that LED light cannot provide an accurate image of blood flow because of its short coherence length [12]. The use of point scanning of illumination potentially also allows three-dimensional tomography of vein structures with the time-domain technique [13].

Recently, an NIR laser and beam expander (line generator lens) have been employed, but these can provide only line scanning on the finger [10]. A micro-vein visualization device using a laser and a Micro Electro Mechanical Systems (MEMS) scanner was also reported for clinical drug injection [14]. However, this device can only detect shallow veins of the forearm skin because it operates in a reflection mode with 740 nm wavelength. Meanwhile, vein identification based on infrared imaging has inherent disadvantages. Since some materials such as carbon ink have a strong tendency to absorb infrared light, it is relatively easy to create a fake vein image [15]. Some cheat trials were also publicized in a technical report under the contract of the Federal Bureau of Investigation (FBI) of the U.S. government [16]. Thus, using blood flow for the detection of liveness can be considered one of the necessary steps for anti-spoofing of finger vein recognition.

In this study, we introduce a new vein imaging method based on a laser and a MEMS scanner (VILS) to overcome the shortcomings of finger vein authentication [11]. The proposed system operates in a transmission geometry, enhancing contrast and allowing liveness. The finger is positioned away from the detection sensor, thereby avoiding any cross-contamination caused by direct contact with the finger. To verify the feasibility of the proposed VILS, finger vein images were analyzed based on the curvature of light intensity and quantitatively compared with those obtained using an LED array. In addition, repeatability tests were carried out to validate the efficacy of the VILS in terms of distinguishability between a live finger and an imitation by detecting blood flow pattern from speckle images of the finger veins.

## 2. Finger Vein Imaging

### 2.1. Operation Principle

**Figure 1:**
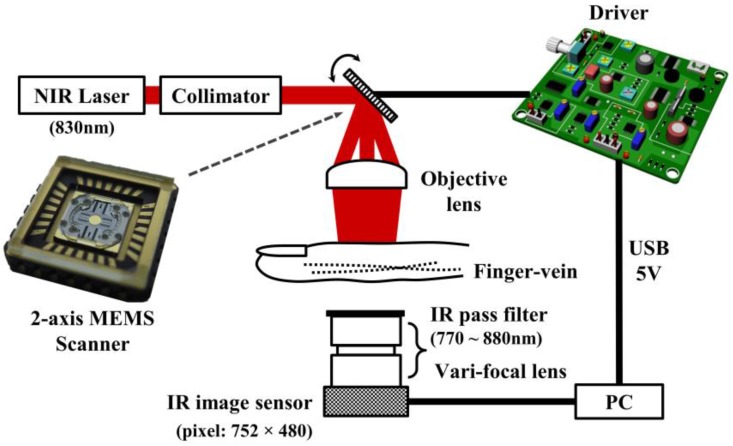
Finger vein imaging system using a near-infrared (NIR) laser and a Micro Electro Mechanical Systems (MEMS) scanner.

The proposed VILS for finger vein imaging is outlined in Figure 1. The NIR laser (830 nm wavelength) is placed in series with the collimator, and the mirror of the MEMS scanner redirects the incident beam toward the objective lens. An index finger is then introduced at the focal position of the objective lens with an effective focal length of 50 mm. The best vein image is obtained when the laser beam is focused on the dorsal surface of the finger. Finally, the NIR beam transmitted through the finger is captured with an IR image sensor. The image of the finger vein pattern taken appears darker than its surroundings because the hemoglobin in the blood vessel absorbs NIR light in the wavelength range of 700–1000 nm [6,7,8,9,10,11].

As shown in the inset of Figure 1, the fabricated 2-axis MEMS scanner is used to scan the finger vein in two dimensions [17]. The electrostatic comb actuation was employed in the MEMS scanner to achieve low power consumption. The MEMS scanner was fabricated by using a self-aligned micro-assembly technique, which does not involve a complicated tilting process. The scanner is designed to operate in a resonance mode along the fast axis, and in a quasi-static mode along the slow axis. The scanning rates of the MEMS scanner were 2 kHz and 20 Hz for the fast and slow axes, respectively. The optical-scan angles were 8.1° and 15.3° for the slow and fast axes, respectively, satisfying the requirement to obtain the minimum necessary image size of 15 mm × 10 mm. Other components included in the experimental setup were an NIR laser (QFLD-830-100S, Qphotonics, Ann Arbor, MI, USA), a collimator (50-850-APC, Thorlabs, Newton, NJ, USA), a MEMS scanner (homemade), an objective lens (PCX 30 × 50 NIR I CTD TS, Edmond Optics, Barrington, NJ, USA), an IR image sensor (FMVU-03MTM-CS, Point Grey, Richmond, BC, Canada), and driver circuits (homemade) for the MEMS scanner and laser. Images of a human finger vein were captured using the IR sensor at a resolution of 480 × 480 pixels, and with 8-bit gray levels. Driver circuits were conveniently operated using the 5 V output from the USB port of a personal computer (PC).

### 2.2. Quantification and Results

**Figure 2:**
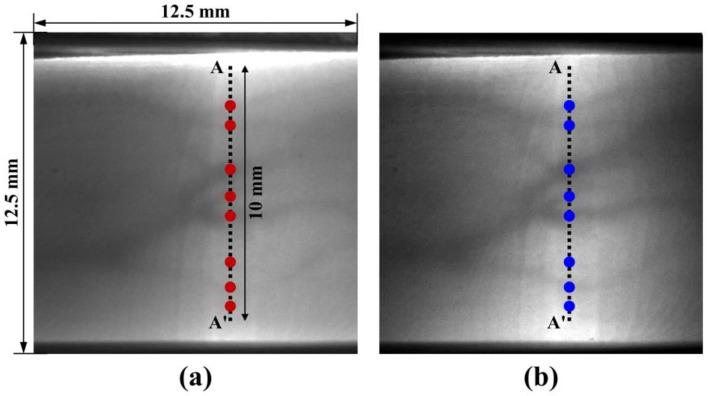
IR sensor images (12.5 mm × 12.5 mm) of an index finger vein using (**a**) the LED array, and (**b**) the laser with the MEMS scanner; the circular dots represent the detected maximum values of the curvature on the vein image.

**Figure 3:**
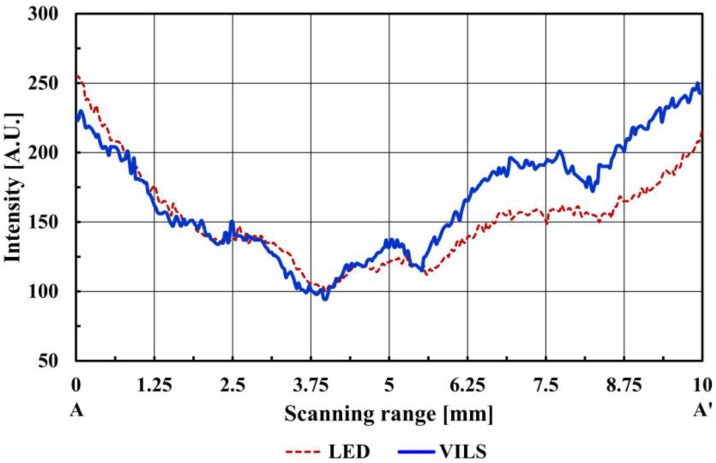
Intensity profile measured along segments A-A’.

Figure 2a,b shows the finger vein images obtained using the LED array (W85I5315-C, Won semiconductor Co. Ltd, Namwon city, Korea) and the laser with the MEMS scanner, respectively. The illumination was carried out with similar optical output powers (LED array: 78.1 mW, and laser: 79.7 mW) and the field of view of the image is 12.5 mm × 12.5 mm. Figure 2b, taken with the laser source, has an apparently higher contrast than Figure 2a, taken with the LED array. To compare the clarity of the two vein patterns quantitatively, the intensity profiles were examined across the segment A-A’ (10 mm in length) for the two types of light sources in Figure 3.

**Figure 4:**
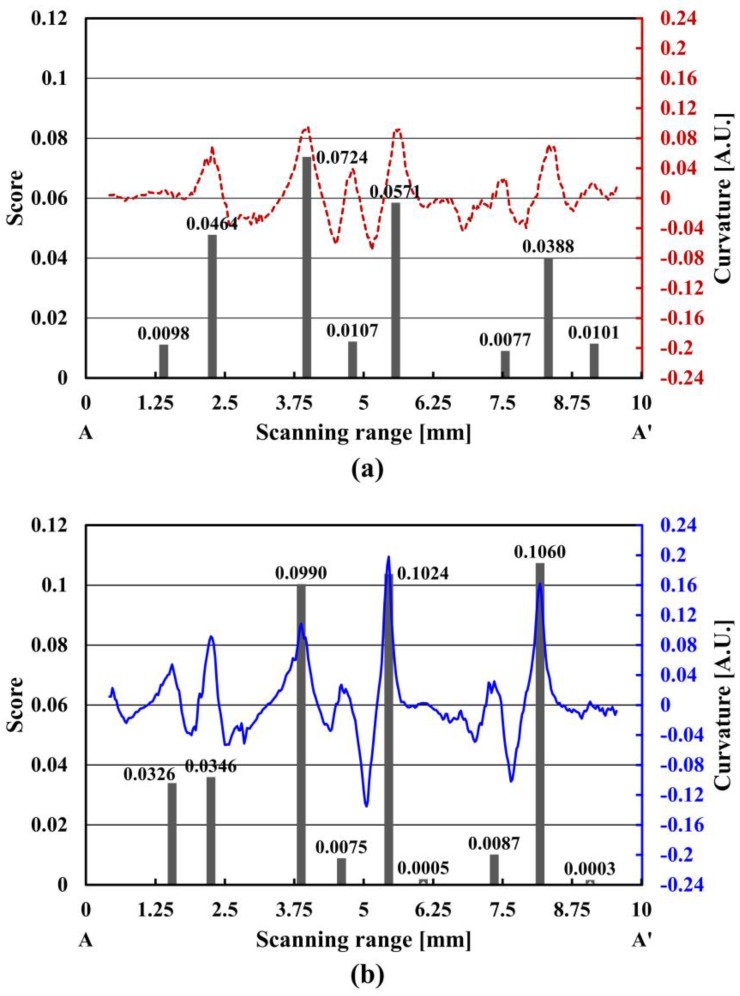
Curvature and score values in the cross-sectional profile for (**a**) the LED array, and (**b**) the VILS.

Prior to the quantitative analysis for finger vein detection, a Gaussian filter was applied across the intensity profile in Figure 3 to reduce the noise that might blur the finger vein positions. The curvature (*κ*) was then calculated to determine the positions and widths of the veins from the local curvature maxima [7,10]. In this study, the one-dimensional curvature in a vertical direction was defined using Equation (1) to evaluate the vein images across the line segment quantitatively.

(1) $$\kappa =\frac{y^{\prime \prime }}{{\left(1+{\left(y^{\prime }\right)}^{2}\right)}^{1.5}},$$

where *y* is the light intensity, *y*’ is its first derivative, and *y*” is its second derivative. To evaluate the magnitude of the finger vein image quantitatively, a score (magnitudes of the finger vein, which is one of the performance indices in vein-pattern recognition) can be defined by Equation (2) [7,10].

(2) $$Score\;=\;P_{c}\;\times \;W_{v},$$

where *P*_c_ represents the local curvature maximum (peak curvature), and *W*_v_ (vein width) is the distance, along the x-axis, between the two nearest zero positions in the curvature plot (Figure 4). The scores were extracted from the curvatures of the filtered intensity by multiplying each peak value with the corresponding width of the positive-curvature lobe.

Table 1 shows the detailed data for the eight finger veins in Figure 4, which shows the curvature maxima in the filtered intensities and widths extracted from the nearest zeros. Considering the two score curves in Figure 4, the threshold score can be determined as 10% of the cumulative density function in the Rayleigh distribution [18]. This criterion will be utilized to find major finger veins in the image analysis. Vein patterns below this threshold were assumed to be either absent or undetectable.

The LED array identified four major finger veins, while the VILS identified five. Four major finger veins (vein numbers: 2, 3, 5 and 7) were identified with both the LED array and VILS, with minor peaks at the other four positions (vein numbers: 1, 4, 6 and 8). The VILS score values are lower than that of the LED at three points (2nd, 4th and 8th). This tendency is not observed consistently in the repeatability test, because the low score levels might be susceptible to external noise. The averaged VILS score for four major finger veins is 60% higher than that of the LED array. This indicates that the VILS is superior to the LED array in terms of image contrast for identity authentication. Note that the scores for the major finger veins, when the crossline perpendicularly intersects the vein pattern, increase by approximately 5.5% compared to those with a skew angle of ±10°.

To perform cross-identification between the users, the obtained finger vein patterns can be utilized in the Scale-Invariant Feature Transform (SIFT) matching method [19,20]. The SIFT features are invariant to the image scaling and rotation because they are local and based on the appearance of the object at particular key points. They are also robust to changes in illumination, noise, and minor changes in the viewpoint. Specifically, the particular key points in the finger vein patterns enable the accurate identification of the correct object.

## 3. Blood Flow Detection

### 3.1. Operation Principle

The authentication capability of the VILS can be further improved by detecting blood flow in finger veins. This technique would serve as an advanced tool to determine whether or not the finger is alive. When coherent laser light is illuminated on a sample area, the light scattered by the medium generates grainy patterns induced by random interference, called laser speckle [12]. If red blood cells flow in the blood vessels, the speckle patterns fluctuate over time. In this respect, laser speckle can be effectively utilized to extract blood flow information [21].

To quantify blood flow, laser speckle images were obtained using the same device as that used to obtain the finger vein images. The magnification of the lens and F-number were adjusted to make speckle size (7.54 μm) approximately equal to the pixel size (6 μm) of the image sensor, so that good speckle images were obtainable for the blood flow detection [21]. The speckle images were captured at 30 frames/s for 1 min. The calculation sequence for extracting blood flow patterns consists of five steps: (1) the total speckle intensity; (2) average speckle intensity (*I*_s_); (3) differences (*I*_d_) between average speckle intensity; (4) averaged difference (*I*_a_); and (5) the ratio (*I*_s_/*I*_a_). The sequence for extracting the blood flow patterns is as follows [21]. First, the total speckle intensity is calculated by summing all pixel values for all the captured images. Second, the average speckle intensity is obtained by dividing the total speckle intensity by the number of frames (*n* = 30). Third, the differences between the average speckle intensity and the captured speckle intensities are calculated. Fourth, the averaged difference is calculated by summing the difference (*I*_d_) and dividing that sum by *n*. Finally, the blood flow image can be obtained as the ratio (*I*_s_/*I*_a_) of the average speckle intensity (*I*_s_) to the averaged difference (*I*_a_).

### 3.2. Blood Flow Image Extraction

**Figure 5:**
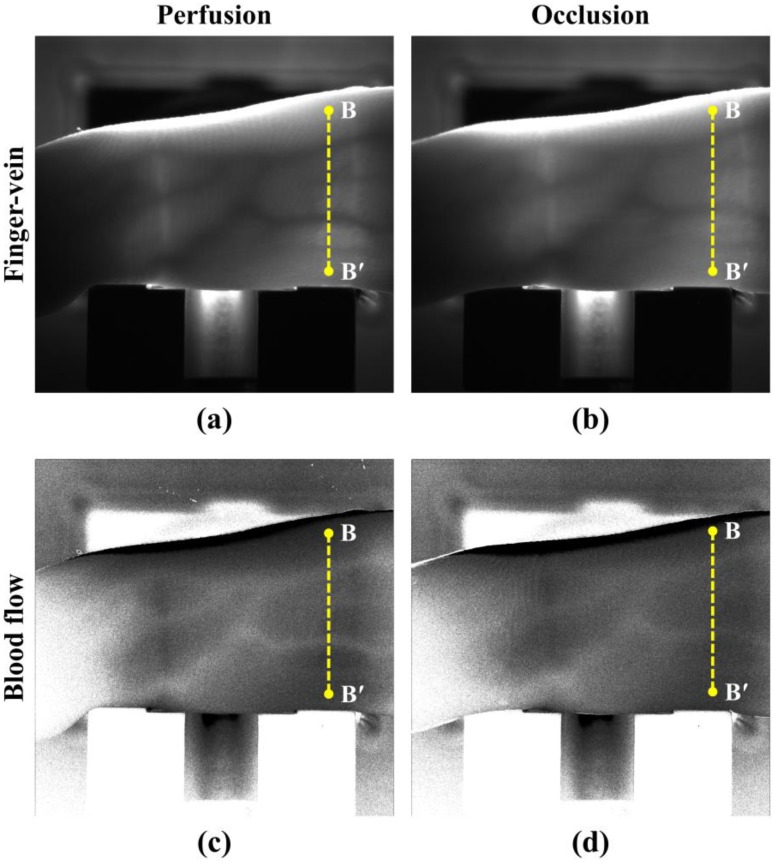
Images of the finger vein in (**a**) perfusion and (**b**) occlusion, and images of the blood flow in (**c**) perfusion and (**d**) occlusion.

To validate the liveness detection capability of the proposed device, the blood flow pattern in perfusion (normal condition) was compared with that in occlusion (artificial condition). Note that these two conditions are analogous to real and imitation fingers, respectively. Perfusion and occlusion were prepared using a sphygmomanometer (arm cuff; pressure: 300 mmHg) to control the blood flow. Figure 5a,b shows finger vein images obtained by averaging the captured speckle images in perfusion and occlusion, respectively. Figure 5c,d shows blood flow images extracted from the speckle fluctuation in perfusion and occlusion, respectively. The brightness level at the vein position is higher than that at the other regions, which contradicts the principle of speckle imaging. This is because the conceptual definition of laser speckle perfusion imaging used in this study is the reciprocal of that of speckle imaging, whose definition is the ratio of deviation and averaged intensity [12,21].

### 3.3. Quantification and Results

**Figure 6:**
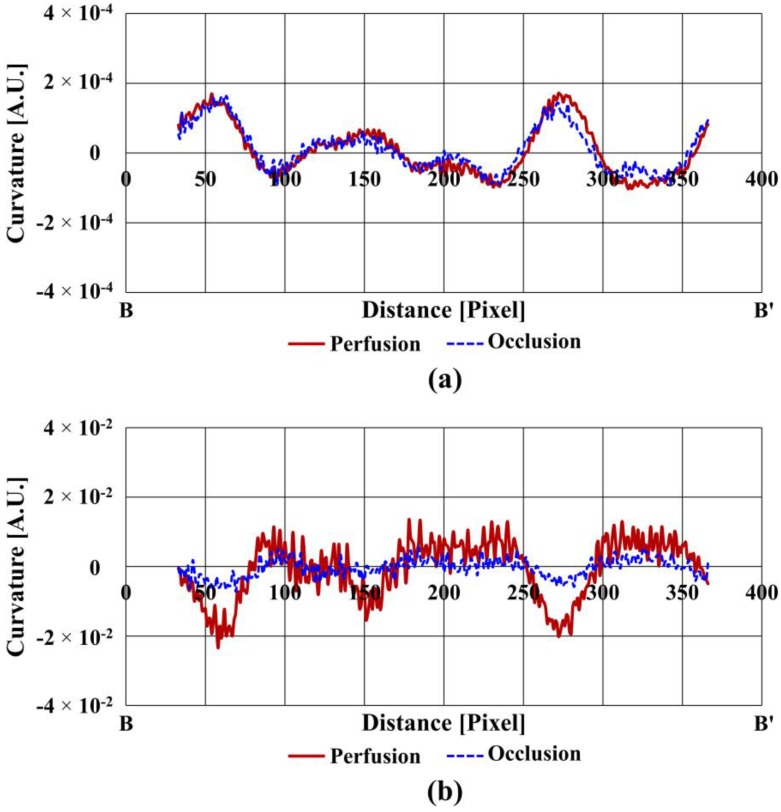
Curvatures of (**a**) finger vein and (**b**) blood flow pattern in the cross-sectional profile (B-B’).

Figure 6a compares the curvatures of the finger vein in two states along the cross-sectional profile (B-B’). Figure 6b shows the curvatures of the blood flow rate (not the amount of blood) under the same condition as that for Figure 6a. The shape of the finger vein curvature in perfusion is similar to that in occlusion, whereas the absolute value (magnitude) of the blood flow curvatures is considerably larger in perfusion compared with that in occlusion, especially at the vein positions. This indicates that blood flow detection can be a powerful method to determine whether a finger is real or an imitation, because occlusion is analogous to the blood flow condition of an imitation finger.

Meanwhile, it is possible to measure blood flow only in perfusion condition in a real finger vein authentication system. Prior to extraction of the quantitative criterion for liveness detection, the sign convention for the blood flow was reversed so that the criterion could be expressed as a positive value. The extraction procedure for the quantitative criterion is as follows. First, the major peak values, which are also located the pixel numbers for the vein positions, were obtained from the finger vein curvature. Second, the peak values were obtained at the same pixel positions in the blood flow curvature. The identical human finger was then repositioned over the IR image sensor three times to test repeatability. Finally, in order to introduce a quantitative criterion for liveness detection, it was required to define a liveness index (*L*_i_) as the peak curvatures of the blood flow (*C*_b_) normalized by those of the finger vein (*C*_v_). This normalization enables the blood flow to remain unaffected by the variation of laser power or variance of finger vein size.

Table 2 shows the normalized blood flow, which is the curvature ratios of the blood flow and finger vein images, at two vein positions in perfusion. I and II in Table 2 represent the peak curvatures of two major finger veins, which is sufficient to detect blood flow, a proxy for liveness. The 1st, 2nd, and 3rd represent the repeatability experiments, carried out by repositioning the human finger. The averaged curvature ratio is 113.2 and its standard deviation is 2.67. It is noticeable that the curvature ratios remain similar to each other regardless of vein size or finger position, which indicates that the curvature value can be used as a criterion for the finger vein authentication system, as is proposed in this study.

## 4. Conclusions

In conclusion, we proposed a novel finger vein imaging device based on a laser and a MEMS scanner (VILS) operating in transmission mode for identity authentication with liveness detection. Quantitative analysis was carried out by calculating the curvature of an intensity-profile after applying a Gaussian filter to a finger vein image. The VILS-based approach demonstrated improved performance by 60% in terms of vein-pattern recognition, compared to an LED array. Liveness detection was validated by extracting speckle-based blood flow in perfusion. The normalized curvature ratio, the peak curvature of the blood flow divided by that of the finger vein, was found, for the first time to our knowledge, to be nearly constant regardless of vein size or finger position. In addition, the significant reliability of the proposed system was confirmed through a repeatability experiment, which indicates that the proposed methodology will improve anti-spoofing authentication performance.

## Figures and Tables

**Table 1:** Performance comparison of the finger vein imaging methods ^1^.

	LED			VILS		
Vein Number	Width (*W*_v_)	Curvature (*P*_c_)	Score (*P*_c_ × *W*_v_)	Width (*W*_v_)	Curvature (*P*_c_)	Score (*P*_c_ × *W*_v_)
1	0.8631	0.0113	0.0098	0.6016	0.0542	0.0326
**2**	**0.6677**	**0.0695**	**0.0464**	**0.3772**	**0.0916**	**0.0346**
**3**	**0.7591**	**0.0953**	**0.0724**	**0.9113**	**0.1086**	**0.0990**
4	0.2739	0.0392	0.0107	0.2773	0.0270	0.0075
**5**	**0.6197**	**0.0921**	**0.0571**	**0.5173**	**0.1979**	**0.1024**
6	0.2909	0.0264	0.0077	0.2765	0.0316	0.0087
**7**	**0.5439**	**0.0713**	**0.0388**	**0.6535**	**0.1622**	**0.1060**
8	0.4911	0.0205	0.0101	0.0594	0.0045	0.0003

^1^ Major finger veins are expressed in bold face.

**Table 2:** Curvature ratios of finger vein image and blood flow image in perfusion at the vein positions.

		Curvature		
Repeatability Test	Vein Positions	Blood flow (*C*_b_)	Finger vein (*C*_v_)	Liveness index (*L*_i_ = *C*_b_/*C*_v_)
1st	I	1.934 × 10^−2^	1.697 × 10^−4^	114.0
	II	2.004 × 10^−2^	1.709 × 10^−4^	117.3
2nd	I	1.770 × 10^−2^	1.546 × 10^−4^	114.4
	II	2.197 × 10^−2^	1.999 × 10^−4^	109.9
3rd	I	1.127 × 10^−2^	1.003 × 10^−4^	112.4
	II	3.233 × 10^−2^	2.916 × 10^−4^	110.9
Average				113.2
